# Epigenetic Alteration by DNA Promoter Hypermethylation of Genes Related to Transforming Growth Factor-β (TGF-β) Signaling in Cancer

**DOI:** 10.3390/cancers3010982

**Published:** 2011-03-03

**Authors:** Sann Sanda Khin, Riko Kitazawa, Takeshi Kondo, Yuka Idei, Masayo Fujimoto, Ryuma Haraguchi, Kiyoshi Mori, Sohei Kitazawa

**Affiliations:** 1 Kobe University Graduate School of Medicine, Division of Diagnostic Molecular Pathology, Kobe 650-0017, Japan; E-Mails: drsanda@gmail.com (S.S.K.); riko@med.kobe-u.ac.jp (R.K.); kondo@med.kobe-u.ac.jp (T.K.); kiyoshim@med.kobe-u.ac.jp (K.M.); idei@kobeh.rofuku.go.jp (Y.I.); marbo-chan@nifty.com (M.F.); 2 Pathology Research Unit, Department of Medical Research (Central Myanmar), Naypyitaw, Union of Myanmar; 3 Ehime University Graduate School of Medicine, Toon 791-0295, Ehime, Japan; E-Mail: ryumaha@m.ehime-u.ac.jp

**Keywords:** methylation, TGF-β signaling, cancer

## Abstract

Epigenetic alterations in cancer, especially DNA methylation and histone modification, exert a significant effect on the deregulated expression of cancer-related genes and lay an epigenetic pathway to carcinogenesis and tumor progression. Global hypomethylation and local hypermethylation of CpG islands in the promoter region, which result in silencing tumor suppressor genes, constitute general and major epigenetic modification, the hallmark of the neoplastic epigenome. Additionally, methylation-induced gene silencing commonly affects a number of genes and increases with cancer progression. Indeed, cancers with a high degree of methylation (CpG island methylator phenotype/CIMP) do exist and represent a distinct subset of certain cancers including colorectal, bladder and kidney. On the other hand, signals from the microenvironment, especially those from transforming growth factor-β (TGF-β), induce targeted *de novo* epigenetic alterations of cancer-related genes. While TGF-β signaling has been implicated in two opposite roles in cancer, namely tumor suppression and tumor promotion, its deregulation is also partly induced by epigenetic alteration itself. Although the epigenetic pathway to carcinogenesis and cancer progression has such reciprocal complexity, the important issue is to identify genes or signaling pathways that are commonly silenced in various cancers in order to find early diagnostic and therapeutic targets. In this review, we focus on the epigenetic alteration by DNA methylation and its role in molecular modulations of the TGF-β signaling pathway that cause or underlie altered cancer-related gene expression in both phases of early carcinogenesis and late cancer progression.

## 1. Introduction

Epigenetics refers to all heritable changes in gene expression and chromatin organization that are independent of the DNA sequence itself. Epigenetic inheritance is an essential mechanism that allows stable propagation of gene activity from one generation of cells to the next [1]. With some exceptions (T-and B-cells of the immune system), all differentiation processes are ultimately triggered and maintained through epigenetic mechanisms [2]. Together with histone modification enzymes, DNA methylation establishes and maintains a condensed or closed chromatin state and the subsequent silencing of gene transcription [3,4]. Indeed, epigenetic changes, especially DNA methylation, have emerged as an important pathway to the development and progression of cancer [5]. A distinguishing feature of epigenetic change, as compared with genetic change, is its reversibility, which makes aberrant DNA methylation an attractive target and offers a good opportunity for the development of epigenetic therapy, diagnosis and prevention in cancer management. Moreover, various environmental and dietary agents and lifestyles are speculated to be partly implicated in the development of human cancers by eliciting epigenetic changes, although the exact molecular mechanisms and precise targets of epigenetic alterations during cancer development are largely unknown.

On the other hand, transforming growth factor-β (TGF-β)/bone morphogenetic protein (BMP) signaling plays a crucial role in a complex and broad biological spectrum including cell proliferation, differentiation, migration, immune response, angiogenesis and apoptosis [6,7]. TGF-β/BMP transduces signals through serine/threonine kinase receptor complexes, which phosphorylate cytoplasmic mediators, the SMADs. Upon phosphorylation, SMADs translocate to the nucleus, associate with transcriptional co-activators or co-repressors and regulate the transcriptional activation of various TGF-β/BMP responsive genes. Moreover, TGF-β/BMP activates cellular mitogen-activated protein kinase (MAPK) signaling pathways, which crosstalk with SMAD signaling, and regulate growth, survival and motility of cells [8]. The role of TGF-β/BMP in cancer development and progression is also complex and often controversial, involving both aspects of tumor suppression and promotion depending on tumor type and stage. In general, TGF-β/BMP signaling initially plays the role of tumor suppressor in epithelial cells, and then of promoter in invasion and metastasis during the later stages of tumor progression. Numerous genetic and epigenetic alterations have been identified in components associated with the TGF-β/BMP signaling pathway that correlate with carcinogenesis, tumor progression and prognosis in various types of malignancies [7-9]. Therefore, a major goal of cancer epigenetic research is to elucidate the molecular mechanisms that relate epigenetic events to carcinogenesis and tumor progression, to reach early diagnosis and to develop prevention as well as novel therapeutic strategies. This review focuses on the epigenetic alterations induced by DNA methylation and the subsequent molecular modulations of the TGF-β signaling pathway, which underlie changed cancer-related gene expression in early carcinogenesis and late cancer progression.

## 2. DNA Methylation and CpG Island Methylator Phenotype (CIMP) in Cancer

Most CpG islands in promoter regions are usually left unmethylated, while some are generally methylated to maintain the inactive X-chromosome and the silenced allele of imprinted genes. Once established, DNA methylation tends to spread and eventually lead to gene inactivation. Although genes that protect against methylation have so far not been identified, CpG islands seem to have developed specific mechanisms to block DNA methylation from spreading [5]. In general, a naked CpG island is unmethylated and coated by proteins that protect against DNA methylation establishment and/or spreading. During repeated rounds of the stem-cell mobilization and replication that accompany ageing, DNA methyltransferases are recruited to the borders of some CpG islands, depositing methyl groups and creating methylation pressure for these islands [10]. This initial recruitment is probably related to repetitive DNA sequences and/or retrotransposons. The balance of methylation pressure and methylation protection may be disrupted in the CpG island methylator phenotype (CIMP), resulting in the spread of methylation into the transcription start area and the triggering of the silencing cascade. The disruption of this balance will probably be achieved through the loss of protective proteins which could occur by mutations that inactivate these proteins or the loss of expression by other mechanisms such as transcription factor loss or histone modifications [11]. Theoretically, this balance could also be disrupted by overactive *de novo* methylation pressure, for example, by activating mutations of DNA methyltransferases. DNA methylation, once established, tends to spread in *cis*, and this spreading is crucial to eventual gene inactivation. The inactivation of a single protecting gene might result in multiple genes being affected simultaneously, exactly as observed in CIMP (CpG Island Methylator Phenotype) [5].

Sporadic tumors with microsatellite instability (MSI), on the other hand, have a heightened frequency of aberrant promoter hypermethylation that affects a number of genes such as CDKN2A, THBSI and MLHI [12, 13]. Furthermore, DNA methylation-associated inactivation of specific genes in colorectal mucosa from the aged population could be one of the earliest events that gives rise to sporadic tumors [2]. Recently, promoter hypermethylation of some genes has clustered in a specific subset of cases termed CIMP (CpG Island Methylator Phenotype), a group of cancers with a 3-5 times higher frequency in aberrant gene promoter hypermethylation. Indeed, 70-80% of sporadic MSI-positive colon cancers are attributed to CIMP and associated MLH1 methylation [13]. Concordant methylation of multiple genes and/or clustering reminiscent of CIMP has been confirmed in colorectal cancer [14,15], bladder cancer [7], renal cell carcinoma [10,16], glioblastoma [17], gastric cancer [18,19], liver cancer [20], pancreatic cancer [21], esophageal cancer [22], ovarian cancer [23], thyroid tumors [24], acute lymphocytic leukemia [25] and acute myelogenous leukemia [26]. In general, the simultaneous methylation of multiple genes, a hallmark of CIMP, is associated with poor outcome in a number of malignancies (head and neck, lung, prostate, esophageal cancer and acute leukemia) [22].

The presence of CIMP signifies the functional and pathophysiological importance of the role of aberrant methylation in cancer, especially that epigenetic changes can be exploited for a novel approach to early diagnosis, prediction of clinical outcome, management and risk assessment [27-29]. Most cancers are curable through surgical resection and adjuvant radiation or chemotherapy if detected early. Unfortunately, with the exception of breast cancer and prostate cancer, early detection screening modalities are invasive and prohibitively expensive [30]. Although reducing promoter hypermethylation of tumor suppressor genes portends a very promising therapeutic target, it may also activate oncogenes in the same cell. Nevertheless, optimized hypomethylation therapies (5-azacytidine and deoxy analogue of 5-azacytidine for the treatment of acute myeloid leukemia (AML) and myelodysplastic syndrome) are desirable and beneficial because some cancers are dependent mainly on silencing tumor suppressor genes for their phenotype and multiple defects. Decitabine (5-aza-2′- deoxycytidine) has been approved for use in liquid tumors, and may be useful in treating solid tumors as well. However, there has been an explosion in the identification and screening of compounds that specifically target other epigenetic components including histone deacetylases and histones methylases. These drugs may be of use both by themselves and in combination with other agents [30]. A central challenge to current cancer therapeutics to improve efficacy is to target specific therapies to genetically or epigenetically distinct tumor types. The potential of molecularly directed therapy, based on targeting the underlying genetic/epigenetic defects, is that it may cause highly selective killing of tumor cells while sparing normal cells, resulting in both increased efficacy and reduced toxicity.

## 3. TGF-β/BMP Signaling and DNA Methylation of Related Genes in Cancer

The TGF-β superfamily comprises more than 40 structurally related polypeptides [31], including TGF-βs, activins and BMPs. Its members play multifunctional and diverse roles in the maintenance of tissue homeostasis by regulating biological processes, including cell growth/differentiation, apoptosis, migration, extracellular matrix formation, inflammatory/immune response, and angiogenesis through heteromeric signaling complexes [6-8,32]. Three TGF-β isoforms, TGF-β1, TGF-β2 and TGF-β3, are expressed ubiquitously in mammalian tissue with TGF-β1 being the predominant one. TGF-β1 is secreted as a latent form in which its active C-terminal remains non-covalently bound to the N-terminal latency-associated peptide (LAP). Before functioning, the captured TGF-β1 must dissociate from LAP through proteolysis, a process known as TGF-β1 activation. TGF-β1 signaling is first elicited when it binds to the TGF-β type II receptor (TGFRII), which in turn activates type I receptor (TGFRI). It then phosphorylates downstream intracellular mediators called SMAD proteins, which include receptor-specific SMADs (R-SMAD,SMAD-2,-3,-5,-8), the common SMADs (Co-SMAD, SMAD-4), and inhibitory SMADs (I-SMADs, SMAD-6 and SMAD-7) [33,34]. Phosphorylated R-SMAD recruits SMAD-4 to form a protein complex, which then translocates into the nucleus and functions as a transcription factor to regulate the expression of TGF-β responsive genes [32,35] and initiates intracellular signaling through mitogen-activated protein kinase (MAPK) pathways. Because of the pleiotropic activities of TGF-β, deregulation of TGF-β signaling has been implicated in numerous pathological conditions, including cancer. Initially, TGF-β signaling is, in general, tumor suppressive in epithelial cells, whereas it promotes invasion and metastasis during the later stages of cancer progression. During tumor progression, however, tumor cells frequently lose the growth-inhibitory response to TGF-β, which is associated with an increased expression of TGF-β in the microenvironment [8,31,36]. Moreover, TGF-β also acts on tumor cells directly and regulates their capacity to remodel the surrounding extracellular matrix (ECM) by enhancing proteinase expression and plasmin generation by the tumor cells. In various cancer cells, TGF-β upregulates the expression of matrix metalloproteinases (MMPs) [37] that activate LAP-TGF-β complex, thereby providing a positive regulatory feedback loop leading to increased TGF-β activation and tumor progression [8].

During tumor progression, tumor cells frequently lose the growth-inhibitory response to TGF-β, and this is associated with an increased expression of TGF-β in the microenvironment. TGF-β-mediated regulation in the tumor microenvironment can be attributed to many factors, including those that involve cell-autonomous signaling, stroma-epithelial interactions, inflammation, immune evasion and angiogenesis. As a multifunctional growth factor, complete abrogation of TGF-β signaling is not a generalized phenomenon in cancers. In fact, in various tumor cells, the TGF-β signaling pathway is functional, and tumor cells can use TGF-β as a tumor-progression factor [38]. As mentioned above, increased production of TGF-β occurs in different tumor types, and correlates with the severity of the tumor grade [39,40]. Tumor-derived TGF-β can affect several cell types in proximity of the tumor, thus producing a microenvironment that promotes tumor growth, invasion and metastasis [8]. TGFβ1-induced inflammation may override its tumor suppressive effect at early stages during carcinogenesis. Moreover, it is worth mentioning that a universal accelerator of DNA methylation is chronic inflammation, as indicated by studied in preneoplastic colon [41], esophagus [42], liver [20] and lung [43].

DNA methylation-associated inactivation of TGF-β-related genes, commonly observed in various human cancers, affects a coordinated decrease in apoptosis, increased proliferation, and decreased differentiation. Epigenetic silencing of human runt-related transcription factor 3 (Runx3) gene (tumor suppressor gene and integral components of TGF-β signaling) expression by promoter hypermethylation plays a critical role in gastric cancer [44], cholangiocarcinoma [45], oral squamous cell carcinoma [46], pancreatic cancer [47], esophageal squamous cell carcinoma [48] and hepatocellular carcinoma [49]. Moreover, suppression of TGF-β and the expression of its receptors by aberrant DNA hypermethylation occurs commonly in renal cell carcinoma [50], head and neck squamous cell carcinoma [51], multiple myeloma [52], lung cancer and prostate cancer [53]. Furthermore, the induction of epithelial-mesenchymal transition (EMT) is accompanied by targeted *de novo* methylation of several gene promoters by TGF-β signaling. The gene silencing via aberrant methylation of all genes which inhibit or facilitates TGF-β signaling, plays important role in human cancers such as breast cancer (EMT genes) [54], ovarian cancer (tumor suppressor FBXO32/SMAD4 target gene) [55], HCC (tristetrapolin (TTP), a negative posttranscriptional regulator of c-Myc) [56], HNSCC (SEPT9, SLC5A8, FUSSEL18, EBF3, and IRX1) [57], cervical cancer [58], desmoids tumor [59] and bladder cancers (BAMBI gene) [7], prostate cancer (SMAD4) [60], malignant pleural mesotheliomas (BMP3b and BMP6) [61], adult T-cell leukemia [62], malignant lymphoma (BMP 6) [63] and gastric cancer (BMP2) [64], *etc.* Given the dynamic epigenetic reprogramming that occurs in cancer cells, DNA methylation profiles observed in human tumors may reflect the history of environmental exposure as does TGF-β signaling during all carcinogenic and progression processes [54].

## 4. Environment-Induced Epigenetic Changes in Cancer

Environmental factors known to play important roles in the etiology of human cancers include chemical carcinogens (e.g., cigarette smoke), dietary contaminants (aflatoxin B1/AFB1) and physical carcinogens (ionizing and UV radiation). Lifestyles such as smoking, alcohol consumption, excess exposure to sunlight, fat consumption and stress may also contribute to cancer development [65,66]. There is ample evidence of an association between DNA methylation and environmental influences like exposure to viruses (e.g., liver [67] and stomach [68]) and diet (e.g., folate) [69] in both normal (aged) tissues and cancer. CIMP-positive tumors are, therefore, also speculated to be related to the environment, lifestyle and genetic predisposition [13]. In general, the degree to which the environment and nutritional factors influence the tumorigenic process depends on the presence of specific hazardous food components, food composition, dietary regime and the amount and duration of exposure. This was observed in our previous epigenetic study of bladder cancer in Myanmar and Japan showing that the rate of gene methylation is different and higher in low-socioeconomic populations [7]. Generally, environmental and dietary/lifestyle factors that are capable of inducing tumor development by eliciting epigenetic changes can be broadly divided into two groups: those that induce direct or indirect changes in genomic DNA, and those that affect critical cellular regulatory processes, such as genes related to transcription, detection of damaged DNA, DNA repair, cell cycle control and cell death. The factors in the first group are exemplified by a study showing that the mismatch repair gene, MHL1, is frequently hypermethylated in sporadic tumors with MSI [12,70]. Similarly, silencing MGMT, the DNA repair gene encoding the protein responsible for the removal of carcinogen-induced O6-methyguanine adducts from DNA (which if left unrepaired results in G to A transition mutation), appears to increase the mutation rate in critical cellular regulators, including tumor suppressors and oncogenes [71,72]. Therefore, exposure to environmental and dietary factors that alter either the expression or the activity of enzymes involved in *de novo* DNA methylation (Dnmt3a and Dnmt3b) and/or in the maintenance of DNA methylation (Dnmt1) may predispose to mutational events [2]. Consequently, CIMP may also be a secondary event to earlier genetic alterations, which ultimately cause increased activity of DNA methyltransferase [11,13]. Alternatively, different agents in the environment and diet may induce mutational events through preferential binding to hypermethylated DNA. This has been shown as a possibility in carcinogenic events through benzo(a)pyrene diol epoxide (BPDE) [73], a carcinogen in tobacco smoke that exhibits preference for methylated CpG sites, and results in the formation of DNA adducts and in G to T transversions often found in cancers of the aero-digestive tract in tobacco smokers [74,75].

Since epigenetic drift, including the loss of global methylation, localized hypermethylation and loss of histone acetylation, occurs gradually, epigenetic changes represent an excellent target for preventive and therapeutic strategies. This is clearly illustrated by studies demonstrating that reduction of DNA methylation prevents the formation of intestinal adenomas in a tumor-prone mouse model [76] and reactivates the expression of genes that have undergone epigenetic silencing [27,77]. While there is accumulating evidence showing that aberrant DNA methylation may result from adverse exposures to epimutagens, there is little evidence regarding the effects of stimuli that cause heritable changes in epigenetic information stored in histones, probably because this is a new and largely unexplored field of cancer epigenetics [2].

## 5. Conclusions

Interest in cancer epigenetics has grown dramatically with the realization that exploiting knowledge of epigenetic changes has tremendous potential for the management of cancer. Epigenetic alterations, as compared with genetic changes, are reversible and typically acquired gradually [2]. New methylation-related findings in cancer and evaluation of hypomethylating drugs *in vitro* and *in vivo*, which have provided approved drugs with few side effects, are helping some patients live longer than those under conventional cytotoxic therapy [78]. Moreover, DNA methylation is proving to be a useful marker of disease risk, activity and prognosis of various malignancies. Despite the complex nature of TGF-β-mediated regulatory signaling in the tumor microenvironment, many aspects of signaling through this pathway have been targeted for therapeutic intervention with the use of systemic and cell-specific strategies that have demonstrated some degree of efficacy [31]. Since epigenetic drift could contribute to the development of chronic diseases including cancer, strategies with different drugs and changes in diet and lifestyle might be highly beneficial in preventing/reversing epigenetic alteration and counteracting the disease. Based on quantitative estimates, over two-thirds of the incidences of cancer are attributed to environmental and dietary factors, making the majority of cancers potentially preventable. Moreover, signals from the microenvironment, especially those from TGF-β signaling, can induce targeted *de novo* epigenetic alterations of cancer-related genes. Since TGF-β deregulation is also partly induced by epigenetic alteration itself, the epigenetic pathway to carcinogenesis and cancer progression has similar reciprocal complexity. In this review, we have discussed the role of DNA promoter hypermethylation of genes related to TGF-β signaling and effects on tumor microenvironment, CIMP and potential therapeutic DNA demethylation machinery and environmental induced epigenetic changes in cancer. Better understandings and the advantages of utilizing aberrant epigenetic information for the discovery of new biomarkers and for the development of novel strategies for cancer prevention will, hopefully, become evident in the near future.
